# Normal-weight obesity is associated with increased risk of subclinical atherosclerosis

**DOI:** 10.1186/s12933-015-0220-5

**Published:** 2015-05-21

**Authors:** Sohee Kim, Chanhee Kyung, Jong Suk Park, Seung-Pyo Lee, Hye Kyoung Kim, Chul Woo Ahn, Kyung Rae Kim, Shinae Kang

**Affiliations:** Department of Internal Medicine, Gangnam Severance Hospital, Yonsei University College of Medicine, 211 Eonju-ro, Gangnam-gu, Seoul, Korea; Severance Institute for Vascular and Metabolic Research, Yonsei University College of Medicine, 211 Eonju-ro, Gangnam-gu, Seoul, Korea; Cardiovascular Center and Department of Internal Medicine, Seoul National University Hospital, 101 Daehak-ro, Jongro-gu, Seoul, Korea; Department of Family Medicine, Health Promotion Center, Gangnam Severance Hospital, Yonsei University College of Medicine, 211 Eonju-ro, Gangnam-gu, Seoul, Korea

**Keywords:** Atherosclerosis, Coronary computed tomography angiography, Fat, Obesity, Pulse wave velocity, Weight, Plaque

## Abstract

**Background:**

Subjects with normal body mass index (BMI) but elevated amounts of body fat (normal-weight obesity; NWO) show cardiometabolic dysregulation compared to subjects with normal BMI and normal amounts of body fat (normal-weight lean; NWL). In this study, we aimed to evaluate whether NWO individuals have higher rates of subclinical atherosclerosis compared to NWL subjects.

**Methods:**

From a large-scale health checkup system, we identified 2078 normal weight (18.5 ≤ BMI < 25 kg/m^2^) subjects with no previous history of coronary artery disease who underwent analysis of atherosclerosis using coronary computed tomography angiography (CCTA) and pulse wave velocity (PWV). NWO was defined as normal BMI and highest tertile of body fat percentage by sex (men ≥25. 4 % and women ≥31.4 %). CCTA was performed using a 64-detector row CT. A plaque was defined as a structure >1 mm^2^ within and/or adjacent to the vessel lumen and classified according to the presence/proportion of intraplaque calcification.

**Results:**

NWO subjects (*n* = 283) demonstrated metabolic dysregulation compared to NWL individuals (*n* = 1795). After adjusting for age, sex, and smoking, NWO individuals showed higher PWV values than NWL individuals (1474.0 ± 275.4 vs. 1380.7 ± 234.3 cm/s, *p* = 0.006 by ANCOVA). Compared with NWL subjects, NWO subjects had a higher prevalence of soft plaques even after age, sex, and smoking adjustment (21.6 % vs. 14.5 %, *p* = 0.039 by ANCOVA). The PWV value and the log{(number of segments with plaque) + 1} showed a positive correlation with numerous parameters such as age, systolic blood pressure, visceral fat, fasting glucose level, serum triglyceride level, and C-reactive protein (CRP) in contrast to the negative correlation with high-density lipoprotein-cholesterol level. The visceral fat was an independent determinant of log{(number of segments with plaque) + 1} (ß = 0.027, SE = 0.011, *p* = 0.016) even after adjustment for other significant factors. Most importantly, NWO was an independent risk factor for the presence of soft plaques (odds ratio 1.460, 95 % confidence interval 1.027–2.074, *p* = 0.035) even after further adjustment for multiple factors associated with atherosclerosis (blood pressure, blood glucose, lipid level, CRP, medication, smoking status, physical activity).

**Conclusions:**

NWO individuals carry a higher incidence of subclinical atherosclerosis compared with NWL individuals, regardless of other clinical risk factors for atherosclerosis.

## Background

Obesity, defined as excess body fat, is a worldwide health problem and a major risk factor for cardiovascular disease [1–3]. Body mass index (BMI) has been used as a parameter of obesity [4]. However, BMI can overestimate the degree of obesity in people with lower body fat percentage and higher lean body mass [5] or underestimate it in Asians with the opposite body composition [6]. Because of these limitations in BMI, subjects with so-called normal-weight obesity (NWO) have been a matter of investigation recently. Specifically, research has focused on whether those with higher amounts of body fat even with normal BMI carry an elevated cardiovascular risk.

In previous studies, NWO subjects have more cardiometabolic risk factors [7–10], impaired cardiac function [11], and are more likely to experience mortality [9] compared to normal-weight lean (NWL) subjects. Furthermore, our group recently demonstrated using ^18^fluorodeoxyglucose-positron emission tomography/computed tomography (^18^FDG-PET/CT) that NWO subjects have more subclinical vascular inflammation [12]. Considering that vascular inflammation is a major pathologic phenomenon leading to plaque rupture [13, 14], these findings suggest that NWO subjects may have more subclinical atherosclerosis, which may in the long-term lead to significant cardiovascular mortality or morbidity. However, no reports have looked into whether NWO subjects are really susceptible to atherosclerosis nor have any in-depth studies investigated the characteristics of atherosclerosis in these patients.

Several methods exist for measuring the degree of atherosclerosis noninvasively. Pulse wave velocity (PWV) is a marker of vascular stiffness, and many reports have shown the association between PWV and cardiovascular events [15]. On the other hand, coronary computed tomography angiography (CCTA) is most useful for visualizing the presence or degree of coronary artery stenosis and is also useful for quantifying plaques and, more importantly, characterizing plaques [16–18].

In this study, we aimed to analyze whether NWO subjects carry a higher incidence of subclinical atherosclerosis than NWL subjects using the above two modalities. The results provided herein demonstrate that NWO individuals carry a higher incidence of subclinical atherosclerosis compared with NWL individuals, regardless of other clinical risk factors for atherosclerosis.

## Methods

### Study design and population

**Fig. 1:**
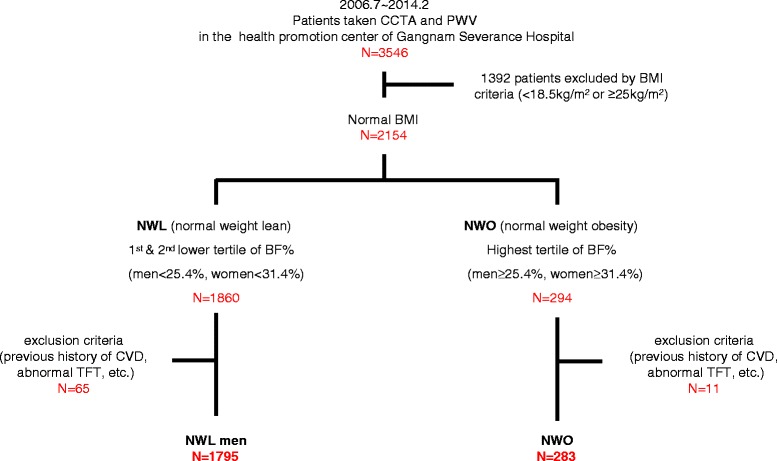
Schematic diagram and flow of the registration. CCTA, coronary computed tomography angiography; PWV, pulse wave velocity; BMI, body mass index; NWL, normal weight lean; NWO, normal weight obesity; BF, body fat; CVD, cardiovascular disease

This cross-sectional study included 3546 consecutive subjects who underwent both CCTA and PWV as part of a self-referred health checkup program at the Gangnam Severance Hospital Health Promotion Center from July 2006 to February 2014. Among the whole population of 3546 subjects, sex-specific tertiles of body fat percentage (BF%) was calculated according to the absolute value of body fat percentage measured by bioelectrical impedance body composition analyzer (BF%, <22.0, 22.0–25.3, ≥25.4 % for men, and <27.3, 27.3–31.3, ≥31.4 % for women). All patients were divided into three groups according to this sex-specific BF% tertiles. Of all subjects, normal weight (18.5 ≤ BMI < 25 kg/m^2^) subjects were the population of interest for the current study (*n* = 2154). Among 2154 normal weight subjects, the population who were in the highest tertile of BF% (BF% ≥25.4 % for men, and ≥31.4 % for women) was defined as NWO and those in the lower two tertiles of BF% (BF% <25.4 % for men, and <31.4 % for women) were defined as NWL. The exclusion criteria of this study were as follows: (1) history of coronary artery disease, (2) significant hypo/hyperthyroidism (thyroid-stimulating hormone >10 μIU/mL or free thyroxine >2.0 ng/dL), and (3) significant renal insufficiency (creatinine >1.5 mg/dL). Ultimately, 2078 subjects (NWL, *n* = 1795; NWO, *n* = 283) were included for analysis in this study (Fig. 1). The protocol of this study was approved by the institutional review board of Gangnam Severance Hospital.

### Anthropometric and biochemical measurements

Data on past medical history, concomitant medication use, smoking, and other medico-social history information were collected through a self-administered questionnaire before the health checkup. The current and past medical history of disease, medication, smoking status (smoker, ex-smoker, non-smoker), alcohol intake (cup/month), salt intake or regular exercise habitus, and other information on medico-social history were self-reported via a routine questionnaire before the health checkup. The definition of ex-smoker was defined as those who quitted smoking for at least 1 month before taking the examination and any subject having a history of smoking within this period was defined as current smoker.

Before anthropometric and biochemical examination, all patients were required to fast for ≥8 h. Height and body weight were measured, and BMI was calculated as weight in kilograms divided by the square of height in meters (kg/m^2^). Total body fat, visceral fat, subcutaneous fat, and body fat percentage were measured and estimated using the bioelectrical impedance body composition analyzer X-Scan Plus II (Jawon Medical, Seoul, Korea). Briefly, the tetra-polar electrode method with 8 touch electrodes was used on both hands and feet, and the bioelectrical impedance value was evaluated by the current intercrossing between each hand and foot. By using the waist-to-hip ratio, body weight, height, age, sex, and the bioelectrical impedance value of the abdominal area, the Jawon Medical Company created a formula specific for Korean individuals to calculate the visceral fat amount. The subcutaneous fat amount was estimated by excluding the visceral fat from the total fat amount and the validity of this method has been extensively tested against CT [12, 19, 20]. Blood pressure (BP) was measured with the patient in a sitting position after ≥15 min of rest. The presence of fatty liver presence was assessed using a Philips iU22 Ultrasound System (Philips Healthcare, Andover, MA, USA). Fasting plasma glucose, total cholesterol, high-density lipoprotein-cholesterol (HDL-cholesterol), triglyceride, calcium, and C-reactive protein (CRP) levels were measured by enzymatic methods using a Hitachi 7600–120 automated chemistry analyzer (Hitachi, Tokyo, Japan). Low-density lipoprotein-cholesterol (LDL-cholesterol) was calculated using the Friedewald formula.

### Coronary artery calcium score, coronary artery stenosis, and coronary plaque measurements using multidetector CT

To measure coronary artery calcium scores (CACS) and coronary artery stenosis and also to characterize intracoronary plaques, CCTA was taken using an adequate multidetector CT scanner (Philips Brilliance 64; Philips Medical System, Best, The Netherlands). We used a standard prospective electrocardiogram-gating protocol with a step-and-shoot technique: 64 × 0.625-mm slice section collimation, 420-ms rotation time, 120 kV tube voltage, and 210 mAs tube current. CACS were calculated with a dedicated software (Extended Brilliance Workspace BW V4.5.2.4031, Philips Medical System, Best, The Netherlands) and expressed as Agatston scores [21].

Based on CACS, the subjects were categorized as having either no coronary calcium (CACS 0 vs. >0) or severe coronary calcium (CACS ≤100 vs. >100). To localize the segment with coronary artery stenosis or plaque, all coronary arteries were categorized as 3 major arteries and 12 small segments. Significant coronary artery stenosis was defined as at least one coronary segment with ≥50 % stenosis. A plaque was defined as a structure >1 mm^2^ within and/or adjacent to the vessel lumen and classified according to the presence/proportion of intraplaque calcification. In detail, plaques were categorized as calcified (calcium [>130 Hounsfield units] content ≥50 % of whole plaque), mixed (calcium content <50 % of whole plaque), and soft (no calcium content). The number of segments with plaque was converted to log scale as log{(number of segments with plaque) + 1}.

### Pulse-wave velocity

Brachial-ankle PWV (ba-PWV) was measured using a volume plethysmographic instrument (VP-1000; Omron Healthcare Corporation, Kyoto, Japan) [22]. The brachial-ankle transit time and distance were calculated as the value of brachial-ankle distance divided by the blood transit time. An automatic device recorded electrocardiograms, phonocardiograms, and BP at both the brachial artery and the posterior tibial artery. Measurements were performed after the patient had rested for ≥5 min in the supine position. The average value of the right and left PWV was used for analysis.

### Statistical analyses

Continuous variables with normal distribution were presented as means ± standard deviation (SD) and those with skewed distribution such as CACS or number of coronary segments with any plaque were log transformed for analysis. Categorical variables were presented as absolute numbers (percentage). Intergroup comparisons were performed using Student’s *t*-test for continuous variables or *χ*2-test for categorical variables as appropriate and also performed using analysis of covariance (ANCOVA) after adjusting for age, sex, and smoking as covariates. The sex- and BMI-matched database was constructed again as necessary by propensity score matching using SAS and the difference of the parameters between NWL and NWO was analyzed by paired *t*-test. The degree of correlation between various cardiovascular risk factors and the parameters of subclinical atherosclerosis, i.e., PWV, coronary artery stenosis, coronary segments with significant stenosis, and coronary plaque, was expressed as the regression coefficients (ß) and standard error (SE) after univariate regression analysis. We performed a multivariate linear regression analysis to identify independent risk factors for subclinical atherosclerosis in normal-weight subjects and the results expressed as ß and SE. Adjusted odds ratios (OR) for the increase in PWV or the presence of coronary plaque in the NWO group compared to the NWL group was estimated with logistic regression analysis models. The SPSS statistical package (version 20.0; SPSS, IBM Corp., Armonk, NY, USA) and SAS macro (version 9.2; SAS Institute, Cary, NC, USA) was used for all statistical analyses. A p-value <0.05 was regarded as statistically significant.

## Results

### Clinical and biochemical characteristics of the study population

Clinical and biochemical characteristics of the study subjects are presented in Table 1. In total, 52.5 % (*n* = 1795) and 8.3 % (*n* = 283) of the whole study population (*n* = 3546) were categorized as NWL and NWO, respectively, after excluding the subjects who did not meet the inclusion criteria. Because NWO subjects were older (p < 0.001) and NWO were less likely to be males (p < 0.001), and have less smokers (*p* = 0.003), we next analyzed all subsequent clinical and biochemical characteristics after adjusting for age, sex, and smoking as covariates using ANCOVA.

**Table 1:** Clinical and biochemical characteristics of the study subjects

	Entire population *n* = 2078	NWL *n* = 1795	NWO *n* = 283	P-value
Age (yr)	53.4 ± 9.2	52.8 ± 9.2	57.6 ± 8.2	<0.001
Male (n, %)	1141 (54.9)	1026 (57.2)	115 (40.6)	<0.001
Smoking status				0.003
Current smoker (n, %)	369 (17.8)	332 (18.5)	37 (13.1)	
Ex-smoker (n, %)	557 (29.1)	494 (27.5)	63 (22.3)	
Non-smoker (n, %)	1152 (55.4)	969 (54.0)	183 (64.7)	
Systolic BP (mmHg)	123.4 ± 15.6	122.5 ± 15.5	128.6 ± 15.7	<0.001
Diastolic BP (mmHg)	77.0 ± 10.1	76.6 ± 10.1	79.6 ± 9.3	<0.001
BMI	22.37 ± 1.7	22.53 ± 4.3	23.9 ± 0.8	<0.001
Body fat percentage (%)	24.4 ± 4.8	23.5 ± 4.3	30.1 ± 3.4	<0.001
Visceral fat (kg)	1.9 ± 0.5	1.75 ± 0.5	2.50 ± 0.4	<0.001
Subcutaneous fat (kg)	12.9 ± 2.5	12.5 ± 2.2	16.0 ± 1.6	<0.001
Fasting glucose (mg/dL)	96.1 ± 17.3	95.7 ± 17.5	99.1 ± 15.9	0.004
Total cholesterol (mg/dL)	193.5 ± 34.8	192.5 ± 34.6	199.6 ± 35.5	0.017
Triglyceride (mg/dL)	107.8 ± 64.9	106.4 ± 65.1	116.6 ± 63.3	<0.001
HDL-cholesterol (mg/dL)	50.8 ± 12.7	51.0 ± 12.8	49.6 ± 11.3	0.002
LDL-cholesterol (mg/dL)	118.0 ± 30.9	117.1 ± 30.6	123.9 ± 32.2	0.002
ALT (IU/L)	22.5 ± 17.35	21.9 ± 17.5	26.4 ± 15.87	<0.001
CRP (mg/L)	1.2 ± 2.0	1.2 ± 2.0	1.6 ± 2.3	0.003
Hypertension (n, %)	697 (33.5)	555 (30.9)	142 (50.2)	<0.001
Diabetes mellitus (n, %)	221 (10.6)	184 (10.3)	37 (13.1)	0.656
BP medication (n, %)	409 (19.7)	319 (17.8)	90 (31.8)	<0.001
Diabetes medication (n, %)	145 (7.0)	118 (6.6)	27 (9.5)	0.677
Lipid lowering agent (n, %)	214 (10.3)	169 (9.4)	45 (15.9)	0.181
Antiplatelet agent (n, %)	223 (10.7)	186 (10.4)	37 (13.1)	0.971
Fatty liver (n, %)	671 (32.3)	531 (29.6)	140 (49.5)	<0.001
Alcohol intake (cup/month)	32.4 ± 11.8	34.0 ± 64.2	22.03 ± 48.4	0.867
Excessive salt intake (n, %)	459 (25.1)	395 (25.0)	64 (25.6)	0.283
Physical activity (n, %)	1432 (73.7)	1245 (74.5)	187 (69.0)	0.049

*NWO* normal weight obesity, *NWL* normal weight lean, *BP* blood pressure, *BMI* body mass index, *HDL-cholesterol* high-density lipoprotein cholesterol, *LDL-cholesterol* low-density lipoprotein cholesterol, *ALT* alanine transaminase, *CRP* C-reactive protein. The difference of age, sex and smoking status between the NWL and the NWO subjects was analyzed with either Student’s *t*-test or *χ*2-test as appropriate. The difference of all other variables was analyzed with analysis of covariance (ANCOVA) using age, sex, and smoking status as covariates. Continuous variables are presented as mean ± standard deviation. Dichotomous variables are presented as the number of subjects with the percentage of subjects in the parenthesis

By ANCOVA, NWO had higher BP (systolic BP, 128.6 ± 15.7 vs. 122.5 ± 15.5 mmHg, p < 0.001; diastolic BP, 79.6 ± 9.3 vs. 76.6 ± 10.1 mmHg, p < 0.001). NWO subjects had greater total body fat percentages and more visceral and subcutaneous fat. In addition, NWO subjects generally displayed an unfavorable metabolic profile compared with NWL subjects. The prevalence of hypertension and the proportion of individuals taking BP medications were higher in the NWO group compared to the NWL group. However, there were no differences in the prevalence of diabetes or the use of antidiabetic, lipid-lowering, or antiplatelet agents between the two groups. Furthermore, there were no differences in nutritional status between the two groups.

### Differences in PWV, coronary calcium, and incidence of intracoronary stenosis/plaque between the NWL and NWO subjects

Compared to the NWL group, the NWO group had a significantly higher mean PWV value even after adjusting with age, sex, and smoking as covariates (1474.0 ± 275.4 vs. 1380.7 ± 234.3 cm/s, *p* = 0.006). However, there were no significant differences in the prevalence of any coronary calcium (CACS >0; 27.2 % vs. 24.3 %, *p* = 0.509), severe coronary calcium (CACS >100; 9.2 % vs. 8.4 %, *p* = 0.286), nor mean difference of log (CACS + 1) (0.43 ± 0.78 vs. 0.39 ± 0.77, *p* = 0.264) between the NWL and NWO groups. There was no difference in the incidence of significant coronary artery stenosis (>50 % stenosis of any major vessel) between the two groups (2.1 % vs. 3.9 %, *p* = 0.075).

Next, we evaluated the prevalence of coronary artery plaque according to the plaque characteristics. The plaques were classified as calcified, mixed, or soft as previously defined. The prevalence of any plaque and the log value of the number of coronary segments with any plaque in the NWO subjects were not significantly different between the two groups (45.2 % vs. 35.6 %, *p* = 0.176 for the prevalence of any coronary plaque; 0.22 ± 0.26 vs. 0.17 ± 0.25, *p* = 0.295 for the log{(number of segments with plaque) + 1}). When the prevalence of plaque was analyzed according to the plaque characteristics, NWO subjects had a significantly higher prevalence of soft plaque (21.6 % vs. 14.5 %, *p* = 0.039) compared to the NWL subjects. However, there were no differences in the incidence of calcified or mixed plaques. Detailed comparisons of the parameters are summarized in Table 2. We also analyzed the difference of the prevalence of soft plaque in the sex- and BMI-matched pairs (*n* = 230 for each group). The PWV value and the prevalence of soft plaque were consistently higher in the NWO subjects compared NWL subjects in the matched pairs (PWV, 1484.3 ± 282.4 vs. 1379.5 ± 251.0 cm/s, p < 0.001; soft plaque, 21.7 % vs. 13.5 %, *p* = 0.026)

**Table 2:** Comparison of various atherosclerosis-associated parameters between the normal weight lean and normal weight obesity subjects

	Entire population *n* = 2078	NWL *n* = 1795	NWO *n* = 283	*P*-value
PWV, (cm/s)	1393.4 ± 242.4	1380.7 ± 234.3	1474.0 ± 275.4	0.006
CACS				
CACS >0 (n, %)	503 (24.7)	427 (24.3)	76 (27.2)	0.509
CACS >100 (n, %)	177 (8.5)	151 (8.4)	26 (9.2)	0.286
Log (CACS +1)	0.40 ± 0.77	0.39 ± 0.77	0.43 ± 0.78	0.264
Coronary artery stenosis (n, %)	76 (3.7)	70 (3.9)	6 (2.1)	0.075
Plaque				
Any plaque (%)	767 (36.9)	639 (35.6)	128 (45.2)	0.176
Log{(Number of segments with plaque) + 1}	0.18 ± 0.26	0.17 ± 0.25	0.22 ± 0.26	0.295
Calcified plaque (%)	358 (17.2)	303 (16.9)	55 (19.4)	0.719
Mixed plaque (%)	295 (14.2)	258 (14.4)	37 (13.1)	0.102
Soft plaque (%)	322 (15.5)	261 (14.5)	61 (21.6)	0.039

*NWO* normal weight obesity, *NWL* normal weight lean, *PWV* pulse wave velocity, *CACS* coronary artery calcium score. Analysis of covariance (ANCOVA) using age, sex, and smoking status as covariates was used to evaluate the difference of continuous variables and dichotomous variables between NWL and NWO subjects. Continuous variables are presented as mean ± standard deviation. Dichotomous variables are presented as the number of subjects with the percentage of subjects in the parenthesis

### Correlation between cardiometabolic factors and PWV or number of coronary segments with plaque

To analyze the risk factors for elevated PWV and the number of coronary segments with plaque, we performed univariate and multivariate regression analyses (Table 3). On univariate analysis, age, systolic and diastolic BP, body fat percentage, amount of visceral fat, amount of subcutaneous fat, and fasting glucose, triglyceride, HDL-cholesterol, and CRP levels were significantly associated with PWV and also with log{(number of segments with plaque) + 1}. To analyze the effect of fat on PWV and the number of coronary segments with plaque, we also performed a multivariate regression analysis with the above significant risk factors. After adjusting for other factors such as age, systolic BP, and fasting glucose, triglyceride, and CRP levels, the amount of visceral fat was an independent risk factor for the number of coronary segments with plaque. However, none of the factors related to fat (body fat percentage and amount of visceral fat or subcutaneous fat) were predictive of PWV after adjusting for other risk factors in a multivariate correlation analysis.

**Table 3:** Degree of correlation between parameters of atherosclerosis and various underlying anthropometric/biochemical parameters

	Univariate regression analysis				Multiple regression analysis	
	PWV, mean		Log(number of segments with plaque + 1)		Log{(number of segments with plaque) + 1}	
	ß(SE)	*P*-value	ß(SE)	*P*-value	ß(SE)	*P*-value
Age	14.040 (0.489)	<0.001	0.011 (0.001)	<0.001	0.010 (0.001)	<0.001
Systolic BP	7.968 (0.292)	<0.001	0.003 (0.000)	<0.001	0.001 (0.000)	<0.001
Diastolic BP	10.014 (0.480)	<0.001	0.004 (0.001)	<0.001		
Visceral fat	72.374 (10.031)	<0.001	0.079 (0.011)	<0.001	0.027 (0.011)	0.016
Subcutaneous fat	1.835 (2.146)	0.393	−0.005 (0.002)	0.021		
Fasting glucose	3.509 (0.297)	<0.001	0.003 (0.000)	<0.001	0.001 (0.000)	<0.001
Total cholesterol	−0.046 (0.153)	0.764	0.000 (0.000)	0.238		
Triglyceride	0.534 (0.081)	<0.001	0.000 (0.000)	<0.001	0.000 (0.000)	0.016
HDL-cholesterol	−2.528 (0.417)	<0.001	−0.004 (0.000)	<0.001		
LDL-cholesterol	0.022 (0.173)	0.900	0.000 (0.000)	0.526		
CRP	15.994 (2.715)	<0.001	0.007 (0.003)	0.013	0.001 (0.003)	0.795

*PWV* pulse-wave velocity, *BMI* body mass index, *BP* blood pressure, *HDL-cholesterol* high-density lipoprotein cholesterol, *LDL-cholesterol* low-density lipoprotein cholesterol, *CRP* C-reactive protein. Univariate and multivariate correlation analysis was performed between the PWV or the number of coronary segments with plaque and the various baseline risk factors enlisted above

*β,* regression coefficient; *SE*, standard error

### Independent risk factors for subclinical atherosclerosis in NWO

To determine the actuarial risk of subclinical atherosclerosis in NWO subjects compared to NWL subjects, we divided the whole population into tertiles according to PWV values. The population was also divided into those with or without coronary plaques and those with or without soft plaques. The risk of being in the highest tertile of PWV was 1.394-fold higher in the NWO group compared to the NWL group after adjusting for age and sex (adjusted OR 1.394, 95 % confidence interval [CI] 1.043–1.862, *p* = 0.025). This turned nonsignificant after further adjusting for systolic BP and fasting glucose, HDL, and CRP levels. Although the risk of having any plaque was higher in NWO compared NWL (adjusted OR 1.494, 95 % CI 1.160–1.924, *p* = 0.002), this also turned out to be nonsignificant after adjusting for age and sex. However, the risk of having a soft coronary plaque in the NWO group was significantly higher than that in the NWL group, even after adjusting for age and sex (adjusted OR 1.586, 95 % CI 1.146–2.194, *p* = 0.005). This importance of NWO subjects having soft coronary plaque was persistently significant, even with the addition of other factors known to influence the development of atherosclerosis, such as systolic BP, fasting glucose, HDL, CRP levels, hypertension medication, diabetes medication, lipid-lowering agents, antiplatelet agents, the smoking status, and physical activity (adjusted OR 1.460, 95 % CI 1.027–2.074, *p* = 0.035) (Table 4).

**Table 4:** Odds ratio of NWO for increased subclinical atherosclerosis

	Odds ratio (95 % CI)		P-value
	NWL	NWO	
	*n* = 1795	*n* = 283	
PWV (highest tertile)			
Model 1	1.000 (Reference)	2.008 (1.557 - 2.591)	<0.001
Model 2		1.394 (1.043 - 1.862)	0.025
Model 3		1.254 (0.927 -1.694)	0.142
Any plaque			
Model 1	1.000 (Reference)	1.494 (1.160 - 1.924)	0.002
Model 2		1.271 (0.954 - 1.693)	0.101
Soft plaque			
Model 1	1.000 (Reference)	1.615 (1.182 - 2.207)	0.003
Model 2		1.586 (1.146 - 2.194)	0.005
Model 3		1.515 (1.084 - 2.116)	0.015
Model 4		1.539 (1.100 - 2.154)	0.012
Model 5		1.555 (1.110 - 2.177)	0.010
Model 6		1.460 (1.027 - 2.074)	0.035

For PWV, the odds ratio for being in the highest tertile of PMV was analyzed. For any coronary plaque or soft plaque, the odds ratio for having these characteristics was analyzed for NWO subjects as compared with the NWL subjects

Model 1 : before adjustment

Model 2 : adjusted for age and sex

Model 3 : adjusted for age, sex, systolic BP, fasting glucose, HDL, CRP

Model 4: adjusted for age, sex, systolic BP, fasting glucose, HDL, CRP, anti-hypertension medication, antidiabetic medication, lipid lowering agent, antiplatelet agent

Model 5: adjusted for age, sex, systolic BP, fasting glucose, HDL, CRP, anti-hypertension medication, antidiabetic medication, lipid lowering agent, antiplatelet agent, smoking

Model 6: adjusted for age, sex, systolic BP, fasting glucose, HDL, CRP, anti-hypertension medication, antidiabetic medication, lipid lowering agent, antiplatelet agent, smoking, physical activity

*CI* confidence interval, *NWO* normal weight obesity, *NWL* normal weight lean, *PWV* pulse wave velocity

## Discussion

In this study, the main findings are as follows: (1) NWO subjects had a higher prevalence of subclinical atherosclerosis. More specifically, NWO subjects were more likely to have soft coronary plaques, independent of other traditional cardiovascular risk factors. (2) NWO subjects had higher risk of having higher PWV value, and also higher risk of the prevalence of having any plaque. (3) The amount of estimated visceral fat itself was an independent risk factor for the above parameters of subclinical atherosclerosis. Altogether, these findings demonstrate that NWO subjects are more prone to carry subclinical atherosclerosis, which may evolve into a significant cardiovascular event in the future.

### Increased prevalence of soft plaque in NWO

Several reports have demonstrated that NWO subjects are more likely to have more cardiovascular risk factors than NWL subjects [7–10]. Although these suggest that these patients are more likely to develop significant future cardiovascular events, there has been a gap of data linking dysregulated cardiometabolic factors and future cardiovascular events. In this aspect, together with a previous study of ours [12], this study demonstrates that NWO subjects do indeed carry a significant degree of subclinical atherosclerosis. More importantly, we have demonstrated more direct evidence that NWO itself is an independent risk factor for having soft plaques, plaques often referred to as “vulnerable” in the literature [23, 24]. Among various plaques, the soft plaque is a powerful predictor of significant cardiovascular events because of its susceptibility to rupture [25]. The fusion of PET and CCTA have identified increased uptake of ^18^FDG in the soft plaque of the coronary artery [26], suggesting a direct link between the characteristics of the plaque and vascular inflammation. These findings also demonstrate the usefulness of employing various imaging tools for identifying those at highest risk for future cardiovascular events.

Although previous studies on the NWO population have consistently shown that NWO patients are indeed “fat,” i.e., they have increased amounts of fat in the body, and that they have elevated systemic inflammatory markers [8, 10, 12, 27], our study showed no significant association between NWO and PWV, CACS, or coronary stenosis. These findings may imply that the important concept of NWO is in identifying the patients with increased vascular inflammation before the progression of vascular stiffness, calcium deposition, or vascular stenosis. Another explanation would be that NWO subjects may be more prone to develop future acute events, as has been shown in a previous report demonstrating that NWO subjects carry a higher risk for cardiovascular mortality [9]. As already known, even with the same “coronary” event, the pathogenesis of stable angina and acute coronary syndrome is completely different, the former being the chronic stable occlusion of the coronary artery by a slowly progressive “stable” plaque causing coronary obstruction and the latter being the acute thrombotic occlusion by plaque rupture [28–30]. Therefore, being an NWO subject may be a strong predictor of the latter.

### No significant association of PWV, CACS, and advanced coronary stenosis in NWO

Although we expected an elevation of PWV, CACS, and advanced coronary stenosis in the NWO subjects, we could not find a significant association between these parameters and NWO. PWV is a relatively simple method to detect the progression of atherosclerosis, and the ESH/ESC guidelines have also recommended PWV as a reliable tool to predict cardiovascular mortality [15, 31]. In our study, the elevated OR for the higher tertile of PWV after adjustment for age and sex in NWO subjects was nonsignificant after further adjustment with cardiovascular disease-related risk factors. This may mean that vascular stiffness might be more influenced by age and sex than other fat-related risk factors, at least in normal-weight subjects [32–34]. On the other hand, this nonsignificant association between PWV and NWO in our study might be because of the different method that we used to measure PWV in our study, ba-PWV and not carotid-femoral PWV [35], and the different study population.

Coronary calcium scoring is another easy method to detect the progression of coronary atherosclerosis. CACS has been shown to predict outcome [36] and it is recommended by the guidelines also that CACS may be reasonable for cardiovascular risk assessment in patients at intermediate risk [37]. However, although CACS may be useful for mass screening, it tells nothing about the composition of the plaque and the additional value of it over the Framingham risk score – which is basically a calculation of the future cardiovascular risk from traditional risk factors – is marginal at best [36]. In addition, a very low or zero CACS does not guarantee ‘zero’ future coronary events [38]. The findings of our study demonstrating that NWO subjects have more soft plaques on CCTA are therefore notable and again demonstrates that NWO subjects should be counted as those at a high risk of future cardiovascular events before the initiation of coronary calcium deposition.

### Stronger association of NWO itself with cardiovascular disease than BMI

Up to now, reports showing that NWO may be associated with future vascular events and cardiovascular mortality have been limited. However, recently, several reports have started to show that BMI might not be a decent predictor of the adverse vascular changes compared to waist circumference or visceral fat [12, 39, 40]. In addition, metabolically abnormal subjects with normal weight seem to exhibit increased subclinical atherosclerosis or vascular inflammation compared to metabolically healthy normal weight or metabolically healthy but obese subjects [41, 42]. In one study, although there was no significant difference in all-cause mortality, cardiovascular mortality was increased in NWO women independent of other cardiovascular disease-related risk factors [9]. In another study, metabolically obese normal-weight subjects — subjects with normal weight but with metabolic dysregulation — also showed higher all-cause and cardiovascular mortality than metabolically healthy normal-weight subjects [41, 43]. The results of those previous papers show that BMI may not be a good predictor of future cardiovascular disease and that a considerable number of normal-weight subjects may be at risk of not being treated properly.

There are some missing links between the metabolically unhealthy normal body weight subjects and future cardiovascular events. Future large-scale, prospective, long-term cohort studies are desperately needed to refute whether these patients are indeed at higher risk for future cardiovascular events. It should also be emphasized that some imaging studies should be complemented as in ours, so as to provide insights into the missing relationship between NWO and cardiovascular events. Furthermore, it will also be important to analyze whether early intervention, either medically or by aggressive lifestyle modification, in NWO subjects would lead to a significant reduction in future events and, if so, who would benefit most by these measures.

## Conclusions

We demonstrated that NWO subjects have a higher subclinical atherosclerosis incidence than NWL subjects, which was largely driven by the higher prevalence of soft plaques in the coronary artery, and that the amount of visceral fat is a major determinant of this subclinical atherosclerosis. A large-scale prospective cohort study might be warranted to prove that reduction of elevated amounts of fat in NWO subjects, by means of diet and/or exercise, will also correct some of the atherosclerotic risk factors in these patients.

## Abbreviations

ALT: Alanine transaminase
BMI: Body mass index
BP: Blood pressure
CACS: Coronary artery calcium score
CCTA: Coronary computed tomography angiography
CI: Confidence interval
CRP: C-reactive protein
HDL: High-density lipoprotein
LDL: Low-density lipoprotein
NWL: Normal-weight lean
NWO: Normal-weight obesity
OR: Odds ratio
PWV: Pulse wave velocity
^18^FDG-PET/CT: ^18^Fluorodeoxyglucose-positron emission tomography/computed tomography

## References

[1] Flegal KM, Carroll MD, Kit BK, Ogden CL (2012). Prevalence of obesity and trends in the distribution of body mass index among US adults, 1999–2010. JAMA.

[2] Flegal KM, Carroll MD, Ogden CL, Johnson CL (2002). Prevalence and trends in obesity among US adults, 1999–2000. JAMA.

[3] Kim MK, Lee WY, Kang JH, Kang JH, Kim BT, Kim SM (2014). 2014 clinical practice guidelines for overweight and obesity in Korea. Endocrinol Metab.

[4] Weisell RC (2002). Body mass index as an indicator of obesity. Asia Pac J Clin Nutr.

[5] Romero-Corral A, Somers VK, Sierra-Johnson J, Thomas RJ, Collazo-Clavell ML, Korinek J (2008). Accuracy of body mass index in diagnosing obesity in the adult general population. Int J Obes (Lond).

[6] Low S, Chin MC, Ma S, Heng D, Deurenberg-Yap M (2009). Rationale for redefining obesity in Asians. Ann Acad Med Singapore.

[7] De Lorenzo A, Martinoli R, Vaia F, Di Renzo L (2006). Normal weight obese (NWO) women: an evaluation of a candidate new syndrome. Nutr Metab Cardiovasc Dis.

[8] Marques-Vidal P, Pecoud A, Hayoz D, Paccaud F, Mooser V, Waeber G (2010). Normal weight obesity: relationship with lipids, glycaemic status, liver enzymes and inflammation. Nutr Metab Cardiovasc Dis.

[9] Romero-Corral A, Somers VK, Sierra-Johnson J, Korenfeld Y, Boarin S, Korinek J (2010). Normal weight obesity: a risk factor for cardiometabolic dysregulation and cardiovascular mortality. Eur Heart J.

[10] De Lorenzo A, Del Gobbo V, Premrov MG, Bigioni M, Galvano F, Di Renzo L (2007). Normal-weight obese syndrome: early inflammation?. Am J Clin Nutr.

[11] Kosmala W, Jedrzejuk D, Derzhko R, Przewlocka-Kosmala M, Mysiak A, Bednarek-Tupikowska G (2012). Left ventricular function impairment in patients with normal-weight obesity: contribution of abdominal fat deposition, profibrotic state, reduced insulin sensitivity, and proinflammatory activation. Circ Cardiovasc Imaging.

[12] Kang S, Kyung C, Park JS, Kim S, Lee SP, Kim MK (2014). Subclinical vascular inflammation in subjects with normal weight obesity and its association with body fat: an 18F-FDG-PET/CT study. Cardiovasc Diabetol.

[13] Libby P (2002). Inflammation in atherosclerosis. Nature.

[14] Davies MJ (1995). Acute coronary thrombosis--the role of plaque disruption and its initiation and prevention. Eur Heart J.

[15] Vlachopoulos C, Aznaouridis K, Stefanadis C (2010). Prediction of cardiovascular events and all-cause mortality with arterial stiffness: a systematic review and meta-analysis. J Am Coll Cardiol.

[16] Hodgson JM, Reddy KG, Suneja R, Nair RN, Lesnefsky EJ, Sheehan HM (1993). Intracoronary ultrasound imaging: correlation of plaque morphology with angiography, clinical syndrome and procedural results in patients undergoing coronary angioplasty. J Am Coll Cardiol.

[17] Assy N, Djibre A, Farah R, Grosovski M, Marmor A (2010). Presence of coronary plaques in patients with nonalcoholic fatty liver disease. Radiology.

[18] Schroeder S, Kopp AF, Baumbach A, Meisner C, Kuettner A, Georg C (2001). Noninvasive detection and evaluation of atherosclerotic coronary plaques with multislice computed tomography. J Am Coll Cardiol.

[19] Ryo M, Maeda K, Onda T, Katashima M, Okumiya A, Nishida M (2005). A new simple method for the measurement of visceral fat accumulation by bioelectrical impedance. Diabetes Care.

[20] Scharfetter H, Schlager T, Stollberger R, Felsberger R, Hutten H, Hinghofer-Szalkay H (2001). Assessing abdominal fatness with local bioimpedance analysis: basics and experimental findings. Int J Obes Relat Metab Disord.

[21] Agatston AS, Janowitz WR, Hildner FJ, Zusmer NR, Viamonte M, Detrano R (1990). Quantification of coronary artery calcium using ultrafast computed tomography. J Am Coll Cardiol.

[22] Jung SJ, Park JH, Lee S (2014). Arterial stiffness is inversely associated with a better running record in a full course marathon race. J Exerc Nutrition Biochem.

[23] Falk E, Shah PK, Fuster V (1995). Coronary plaque disruption. Circulation.

[24] Falk E (1983). Plaque rupture with severe pre-existing stenosis precipitating coronary thrombosis. Characteristics of coronary atherosclerotic plaques underlying fatal occlusive thrombi. Br Heart J.

[25] O’Holleran LW, Kennelly MM, McClurken M, Johnson JM (1987). Natural history of asymptomatic carotid plaque. Five year follow-up study. Am J Surg.

[26] Alexanderson E, Slomka P, Cheng V, Meave A, Saldana Y, Garcia-Rojas L (2008). Fusion of positron emission tomography and coronary computed tomographic angiography identifies fluorine 18 fluorodeoxyglucose uptake in the left main coronary artery soft plaque. J Nucl Cardiol.

[27] Berg AH, Scherer PE (2005). Adipose tissue, inflammation, and cardiovascular disease. Circ Res.

[28] Davies MJ (2000). The pathophysiology of acute coronary syndromes. Heart.

[29] Moreno PR, Falk E, Palacios IF, Newell JB, Fuster V, Fallon JT (1994). Macrophage infiltration in acute coronary syndromes. Implications for plaque rupture. Circulation.

[30] Motoyama S, Sarai M, Harigaya H, Anno H, Inoue K, Hara T (2009). Computed tomographic angiography characteristics of atherosclerotic plaques subsequently resulting in acute coronary syndrome. J Am Coll Cardiol.

[31] Hypertension EETFftMoA (2013). 2013 Practice guidelines for the management of arterial hypertension of the European Society of Hypertension (ESH) and the European Society of Cardiology (ESC): ESH/ESC Task Force for the Management of Arterial Hypertension. J Hypertens.

[32] Davies JI, Struthers AD (2003). Pulse wave analysis and pulse wave velocity: a critical review of their strengths and weaknesses. J Hypertens.

[33] Jani B, Rajkumar C (2006). Ageing and vascular ageing. Postgrad Med J.

[34] Im JA, Lee JW, Shim JY, Lee HR, Lee DC (2007). Association between brachial-ankle pulse wave velocity and cardiovascular risk factors in healthy adolescents. J Pediatr.

[35] Ito N, Ohishi M, Takagi T, Terai M, Shiota A, Hayashi N (2006). Clinical usefulness and limitations of brachial-ankle pulse wave velocity in the evaluation of cardiovascular complications in hypertensive patients. Hypertens Res.

[36] Greenland P, LaBree L, Azen SP, Doherty TM, Detrano RC (2004). Coronary artery calcium score combined with Framingham score for risk prediction in asymptomatic individuals. JAMA.

[37] Greenland P, Alpert JS, Beller GA, Benjamin EJ, Budoff MJ, Fayad ZA (2010). 2010 ACCF/AHA guideline for assessment of cardiovascular risk in asymptomatic adults: a report of the American College of Cardiology Foundation/American Heart Association Task Force on Practice Guidelines. J Am Coll Cardiol.

[38] Doherty TM, Wong ND, Shavelle RM, Tang W, Detrano RC (1999). Coronary heart disease deaths and infarctions in people with little or no coronary calcium. Lancet.

[39] Ren C, Zhang J, Xu Y, Xu B, Sun W, Sun J (2014). Association between carotid intima-media thickness and index of central fat distribution in middle-aged and elderly Chinese. Cardiovasc Diabetol.

[40] Lukich A, Gavish D, Shargorodsky M (2014). Normal weight diabetic patients versus obese diabetics: relation of overall and abdominal adiposity to vascular health. Cardiovasc Diabetol.

[41] Yoo HJ, Hwang SY, Hong HC, Choi HY, Seo JA, Kim SG (2014). Association of metabolically abnormal but normal weight (MANW) and metabolically healthy but obese (MHO) individuals with arterial stiffness and carotid atherosclerosis. Atherosclerosis.

[42] Yoo HJ, Kim S, Hwang SY, Hong HC, Choi HY, Seo JA (2015). Vascular inflammation in metabolically abnormal but normal-weight and metabolically healthy obese individuals analyzed with (1)(8)F-fluorodeoxyglucose positron emission tomography. Am J Cardiol.

[43] Choi KM, Cho HJ, Choi HY, Yang SJ, Yoo HJ, Seo JA, Kim SG, Baik SH, Choi DS, Kim NH. Higher Mortality in Metabolically Obese Normal Weight People than in Metabolically Healthy Obese Subjects in elderly Koreans. Clinical endocrinology 2013 10.1111/cen.12154 23330616

